# Supplementary material for: Stacking Model‐Based Classifiers for Dealing With Multiple Sets of Noisy Labels
Source: Biom J. 2025 Mar 12;67(2):e70042. doi: 10.1002/bimj.70042 (PMC11898607; doi:10.1002/bimj.70042)
Supplement: Supplementary file 1 — Supporting Information [file BIMJ-67-e70042-s001.zip › density-based-ensemble-model-main/competitors_real_data/netlab-master/netlab-master/help/rbfpak.htm]

Netlab Reference Manual rbfpak


# rbfpak

## Purpose

Combines all the parameters in an RBF network into one weights vector.

## Synopsis

```
w = rbfpak(net)
```

## Description

`w = rbfpak(net)` takes a network data structure `net` and combines
the component parameter matrices into a single row vector `w`.

## See Also

`rbfunpak`, `rbf`

---

**Pages:**
Index


---

Copyright (c) Ian T Nabney (1996-9)
